# Influence of personalized therapeutic approach on quality of life and psychiatric comorbidity in patients with advanced colonic cancer requiring palliative care

**Published:** 2010-08-25

**Authors:** O Popa–Velea, B Cernat, A ţambu

**Affiliations:** * Lecturer, ‘C.Davila’ University of Medicine and Pharmacy, Department of Medical Psychology, BucharestRomania; ** Student, ‘C.Davila’ University of Medicine and Pharmacy – BucharestRomania

**Keywords:** quality of life, personalized approach, colonic cancer, anxiety, depression

## Abstract

Personalized medical care has been consistently proven in literature as contributing to the maintenance of psychological balance and quality of life in patients suffering from chronic conditions. However, limited research has investigated the role of personalized approach in improving these parameters in patients with advanced incurable diseases. The scope of this paper was to investigate the possible impact of personalized care condition in advanced colon cancer patients, requiring palliative care. 60 patients (32 M, 28 F) (mean age 64,6) suffering from this disease were randomly assigned to a standard or to a personalized care condition. The latest implied (a) frequent (at least 2 monthly) meetings with the doctor, (b) possibility to be involved in treatment decisions, (c) more information given about diagnosis and prognosis and (d) psychological support provided to the patient and his/her family members, to deal better with daily problems and needs. The design of the study was prospective and consisted of two successive evaluations of quality of life (SF–36 questionnaire) and anxiety and depression (HAD test). Cancer patients pertaining to the personalized treatment approach had both superior quality of life scores (p < 0,05) and lower anxiety (p < 0,01) and depression (p < 0,05) than the control group. A more detailed analysis showed significant differences of vitality and social functioning for subjects pertaining to the study group (p < 0,05), as well as a lower ratio between  latent and manifest anxiety (p < 0,01). These results argue in favor of the benefits of a personalized treatment approach for patients with advanced incurable diseases.

## Background

Colonic cancer is one of the most important health problems in developed countries, being placed on a leading 3^rd^ position (after stomach and lung neoplasms) among the most common forms of cancer [1]. 

The advanced form of colonic cancer is considered the condition where at presentation the cancer is either metastatic or so locally advanced that surgical resection is unlikely to be carried out with a curative intent. Typically, a combination of palliative therapeutic resources (chemotherapy, radiotherapy and conservative surgery) is used in this case, but all these techniques are likely to affect the quality of life of patients, via significant side effects and / or emerging lifestyle restrictions [2]. In this context, the quality of the doctor–patient relationship can become a key factor for prognosis: it can moderate or, reversely, contribute to further depreciation of patients' quality of life, and, in turn, it can influence in both ways the adherence to the treatment.

Studies done so far with this respect in oncology [3, 4] showed that involving the patient in medical decisions (within a so–called ‘personalized therapeutic plan’) is a potent vector that can maintain or even enhance one's quality of life. 

Primary argument in favor of this position is that cancer patients have an immediate perspective of their own suffering and death; therefore, they should not only have the right to know the diagnosis, but also to be part of all critical treatment decisions [5, 6]

Inversely, other authors claim that especially in situations where life expectancy is uncertain and efficacy of therapeutic measures is doubtful, the physician should be the only one to take the primary decisions [7]. The proponents of this view argue that information delivery, deliberation and decision taking are classically assumed only by the doctors and offering opportunities of decisions to patients would just contribute to their confusion [8,9, 10].

So far, advocates of the first position seem to be backed-up by more data, at least in the case of colonic cancer [11]. However, the implementation in practice of personalized therapeutic plans is still rather rare; therefore, studies done so far on this topic are also rare and far from conclusive.

## Objectives

The aim of this study was to evaluate the impact of a personalized therapeutic plan on the quality of life and psychological symptoms (anxiety, depression) for patients suffering from advanced colonic cancer and requiring palliative care. 

Our hypotheses consisted in arguing in favor of:

- a positive effect of this plan on the quality of life of the patient (despite the multitude of physical symptoms brought by the disease);
- a lower vulnerability to psychiatric comorbidity (anxiety, depression) in patients attending a personalized therapeutic plan.

## Materials and methods

### Participants

The study's number of participants was 60 patients (32 males and 28 females) (mean age = 64,6; SD = 3,2) with advanced colonic cancer in the post–colectomy phase and who had a stable relationship with their oncologist in the previous two months before testing.

Control group (n = 30) (15 males and 15 females) (mean age = 62,7; SD = 3,1) was represented by patients carrying a chronic non–malignant, well tolerated condition (arterial systemic hypertension or diabetes mellitus). 

Three doctors were involved in the study (two for the study group and one for the control group), with no differences between them regarding the age, location and expertise. 

### Inclusion and exclusion criteria 

We included in the study only patients with advanced colonic cancer (stage 3 B), in the post–surgical phase (after colectomy with colostomy), who had a stable relationship with their doctor for the last two months, were not treated by other doctors or health professionals, and consented to be subjects of this study for a 8–week continuous period. Patients who could not fulfill the above criteria, those with somatic co–morbidities that could affect their quality of life, and / or with neuropsychiatric disorders that could affect their responsibility (e.g. psychoses, Alzheimer) were not admitted in the study.

### Method Design

The design of the study was prospective. Patients from both groups (colonic cancer and control) were randomly assigned to a standard or to a personalized care condition, the latest implying four distinct features:

- frequent meetings with the doctor (at least  two monthly);
- possibility of the patient to be actively involved into treatment decisions;
- more information given about the therapy and prognosis;
- psychological support provided to the patient and his / her family members, to deal better with the daily problems and needs.

Participants were tested at the beginning of the study and after a two–month period, in which they had at least 3 meetings with their doctor. The variables tested were:

- quality of life;
- anxiety;
- depression;
- the degree of personalization of the doctor–patient relationship (i.e. appropriateness to the patient's needs), as seen by the patient.

Data were collected into a database using the SPSS 16.0 software. The differences between scores at the entrance and scores at the end of the study for first three study variables in relationship to the fourth one were computed and statistically evaluated. Comparisons of results were done, using multivariate analysis of variance and paired samples t–tests. Influence of age and gender was controlled. All results were considered significant at a threshold of p < .05.

### Instruments

1. SF–36 Quality of Life (QoL) Questionnaire [12] It is the most widely used instrument for the measurement of the quality of life (QOL). It is translated into numerous languages and the validity of the 8 subscales is confirmed in general populations and in a wide variety of patient groups in more than 2000 articles. It comprises 36 questions and 8 scales, dedicated to self–perceived functional health, general well–being, and to various components of physical and mental health (such as pain perception or vitality). Scores are reported on a scale from 0–100 (mean = 50, SD = 10), and are proportional to QoL.
2. HAD (Hospital Anxiety and Depression Scale) [13]It is a simple 14–items test, to evaluate anxiety and depression in hospital settings. Each item is answered by the patient on a four point (0–3) response category, with scores of 11 or higher – for either subscale – indicating probable presence of a mood disorder and a score of 8 to 10 being just suggestive of the presence of the respective state.
3. An original questionnaire (8 questions) (appendix 1) Was designed to evaluate the degree of personalization of the relationship, as it is perceived by the patient. Answers were coded numerically (1...4 or 1...5), with scores proportional to the degree of personalization of the approach. The range of answers was 8 to 39; scores above 26 (upper 1/3) were considered relevant for a personalized approach.

## Results

Not surprisingly, the components of the quality of life were different across clinical conditions, with consistently lower SF–36 scores obtained by cancer patients:[Table 1]

**Table 1:** Comparison of SF–36 components scores, across clinical conditions (ANOVA)

SF–36 scale	F	p
General health	46,17	.01
Ability to perform physical roles	25,67	.01
Vitality	19,66	.01
Physical functioning	16,52	.05
Body pain	18,16	.05
Social functioning	15,15	.05

However, even if the quality of life was lower inside the two cancer groups, patients attended in a personalized manner had significantly higher scores of vitality and social functioning (p < 0,05), compared to cancer patients who were subject to standard care[Table 2]:

**Table 2:** Quality of life (mean, range) across care conditions (* F = 13,74, p < .05 (ANOVA); ** F = 14,15, p < .05 (ANOVA))

SF–36 scales	Cancer patients (n = 60)		Non–cancer patients (n = 30)	
	Personalized	Standard	Personalized	Standard
Physical functioning	66,7 (61,3–74,7)	64,9 (58,6–68,8)	76,9 (72,2–79,6)	73,3 (64,7–76,1)
Ability to perform physical roles	55,8 (53,3–59,1)	58,6 (49,9–61,7)	71,5 (67,7–74,8)	72,6 (68,9–74,9)
Bodily pain	65,6 (62,3–66,8)	69,6 (61,1–77,5)	79,3 (74,8–81,7)	76,2 (66,5–84,3)
General health	63,5 (61,1–67,8)	62,4 (56,3–69,1)	75,6 (71,2–80,1)	77,3 (71,6–84,7)
Vitality	68,3* (62,9–71,1)	51,3* (48,6–54,3)	74,5 (68,1–79,9)	72,1 (68,3–74,6)
Social functioning	72,3** (70,2–80,4)	64,5** (62,8–66,9)	86,2 (87,7–89,7)	88,1 (80,9–91,3)
Ability to perform emotional roles	76,4 (70,9–81,7)	75,1 (66,4–84,2)	79,4 (71,5–86,1)	78,4 (72,1–85,2)
Mental health	84,8 (79,4–90,1)	80,5 (74,6–89,2)	85,3 (80,2–90,8)	85,1 (79,7–92,2)

**Figure 1:**
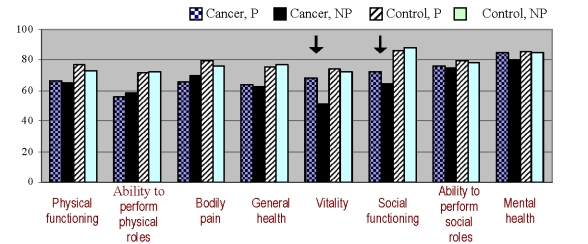
Quality of life (mean, range) across care conditions

Inside the cancer group, the global quality of life scores were also different by approach (F = 19,07, p < .05). Still, the effect was not significant for people pertaining to the control group (F = 3,24, ns). This finding illustrates the comparative higher importance of approach type for the advanced cancer patients.

Anxiety and depression scores were inversely correlated to the scores of the questionnaire, assessing the degree of personalization of the doctor-patient relationship (t = 4.55, p < .02 and t = 2.86, p < .05, respectively). In other terms, this means that, as the doctor–patient relationship was perceived as more tailored to patient's needs, there were fewer chances that after a period of 2 months the patient developed anxiety and / or depression.

## Discussions

Although the quality of life was largely dependent on the disease (with lower scores associated to the advanced cancer condition), the type of doctor–patient relationship was able to influence significantly at least two of its components (vitality and social functioning). For both, the personalized approach proved to be more advantageous. This effect was met in both the study group and controls, but was statistically significant only in cancer patients.

In terms of anxiety and depression, the perception of the doctor's approach as tailored to patient's needs was significantly associated with a lower risk of occurrence of these clinical conditions. This effect was obtained rather quickly, after only two months of sustained better doctor–patient communication.

Both findings are important, as care is still often oriented to advanced cancer and more to easing the physical suffering and offering support to a merely passive patient, and not to maintain the patient as an active participant in the decisional process [14, 15].

Maintaining vitality and social functioning in cancer can have in turn a direct effect on the disease, as it offers the prospect of a longer life expectancy, via enhancing the patient's resources to cope and to conserve his / her ability to have an active social life. Lower levels of anxiety and depression can contribute to the preservation of immunity capacities and to therapeutic adherence [16, 17], thereby also improving the patient's prognosis. 

The limits of the study were the low number of participants, incertitude about the possible social desirability effects and not taking into account some other relevant variables (e.g. personality type). Further research could provide more refined data in this matter; however, these preliminary results could be an argument in favor of the benefits of a personalized treatment approach for patients with advanced oncological illnesses.

## Appendix 1

Questionnaire evaluating the degree of personalization of the doctor–patient relationship, as it is perceived by the patient

Please answer the following 8 questions, by expressing honestly of your thoughts and your reflections about the treatment and the relationship with your current physician. The information you provide will be treated with confidentially and will be used only for research purposes

1. The information about the disease that was provided to you by the doctor is:
  1. not satisfactory (I don't really know which is the disease I suffer from);
  2. somehow satisfactory, but not sufficient;
  3. satisfactory, but I would like to know more;
  4. almost completely satisfactory;
  5. completely satisfactory (I know enough details about my disease).
2. You received explanations regarding your disease:
  1. not at all;
  2. quite a few;
  3. a few;
  4. sufficient;
  5. sufficient and comprehensive (I could understand better what I am suffering from)
3. Were you ever asked if you would like to participate in treatment decisions?
  1. No, never, but I wouldn't like to;
  2. No, but I wouldn't mind;
  3. Yes, but I didn't like to be part of;
  4. Yes, and I participated in such decisions.
4. Did you get any explanations about the treatment itself?
  1. not at all;
  2. quite a few;
  3. a few;
  4. sufficient;
  5. sufficient and comprehensive (I could understand better the treatment I got)
5. You got the main information about the disease from:
  1. friends and / or neighbors;
  2. family;
  3. some person I know, who works in the medical field;
  4. the nurse;
  5. my current physician.
6. Your relationship to the medical staff that cares for you is:
  1. very distant;
  2. somehow distant;
  3. average;
  4. rather positive;
  5. very positive.
7. The relationship to your family / relatives is:
  1. very distant;
  2. somehow distant;
  3. average;
  4. rather positive;
  5. very positive
8. What is your opinion about your doctor's capacity to handle your disease?
  1. I am very pessimistic;
  2. I am rather skeptical;
  3. I am not sure, somewhere in the middle;
  4. I am somehow optimistic;
  5. I am optimistic
